# *In Vivo* Small Molecule Delivery to the Optic Nerve in a Rodent Model

**DOI:** 10.1038/s41598-018-22737-4

**Published:** 2018-03-13

**Authors:** Shandiz Tehrani, R. Katherine Delf, William O. Cepurna, Lauren Davis, Elaine C. Johnson, John C. Morrison

**Affiliations:** Casey Eye Institute, Department of Ophthalmology, Oregon Health & Science University, Portland, OR USA

## Abstract

Small molecule delivery to the optic nerve would allow for exploration of molecular and cellular pathways involved in normal physiology and optic neuropathies such as glaucoma, and provide a tool for screening therapeutics in animal models. We report a novel surgical method for small molecule drug delivery to the optic nerve head (ONH) in a rodent model. In proof-of-principle experiments, we delivered cytochalasin D (Cyt D; a filamentous actin inhibitor) to the junction of the superior optic nerve and globe in rats to target the actin-rich astrocytic cytoskeleton of the ONH. Cyt D delivery was quantified by liquid chromatography and mass spectrometry of isolated optic nerve tissue. One day after Cyt D delivery, anterior ONH filamentous actin bundle content was significantly reduced as assessed by fluorescent-tagged phalloidin labeling, relative to sham delivery. Anterior ONH nuclear counts and axon-specific beta-3 tubulin levels, as well as peripapillary retinal ganglion cell *layer* nuclear counts were not significantly altered after Cyt D delivery relative to sham delivery. Lastly, the surgical delivery technique caused minimal observable axon degeneration up to 10 days post-surgery. This small molecule delivery technique provides a new approach to studying optic neuropathies in *in vivo* rodent models.

## Introduction

Optic neuropathies such as glaucoma^1^ result from injury to optic nerve axons at the level of the optic nerve head (ONH)^2–7^. The mechanism of glaucomatous axon injury is likely multifactorial, and may involve biomechanical strain^8–14^, immune-modulated inflammatory responses^15–21^, protective or deleterious contributions from reactive astrocytes^22–29^, and altered vascular perfusion^30–34^. However, definitive insight into the early sequence of cellular events that lead to eventual axon injury is lacking. In particular, the question of how elevated intraocular pressure (the only known modifiable risk factor for glaucoma) leads to eventual axon injury remains unanswered. Lastly, targeted axon-specific therapies for glaucoma are lacking^29,35^.

Small molecule delivery to the ONH would allow for probing of potential cellular and molecular pathways involved in axon injury in previously established animal models of glaucoma^3,36–50^. Furthermore, small molecule delivery to the ONH would allow for screening of novel and experimental therapeutics that target the ONH in animal models of glaucoma. Previous attempts to deliver small molecules to the intact optic nerve in rodent and non-human primate models included the use of osmotic minipumps implanted subcutaneously near the orbit and connected to a tube surgically implanted through the sub-Tenon’s space into the retrobulbar space^51–53^. In addition, direct optic nerve visualization through a surgical approach in a rat model, followed by placement of a surgical sponge (soaked in a small peptide solution) along the exposed optic nerve, has been reported^54^. However, the above techniques did not include surgical dissection of the optic nerve sheath to allow for optimal delivery of small molecules to the optic nerve neural tissue, and delivery of the small molecules to the optic nerve tissue was not quantitatively confirmed through analytical testing. Direct delivery of small molecules (namely short interfering RNA) to the optic nerve stump after optic nerve transection in a rat model has been reported, and further confirmed by down regulation of the target protein level^55^.

Here, we describe a surgical method for small molecule delivery to the optic nerve and ONH in a rat model, and quantitatively confirm delivery of small molecules to the optic nerve tissue using liquid chromatography and mass spectrometry (LC/MS). This technique was further optimized for small molecule delivery to the optic nerve neural tissue by including an optic nerve sheath fenestration step. Lastly, the effect of small molecule delivery on the optic nerve head and peripapillary retina was assessed. Our proof-of-principle technique involved delivery of the small molecule cytochalasin D (Cyt D), which is a cell-permeable inhibitor of filamentous actin polymerization and reduces total filamentous actin^56^. The ONH is rich in filamentous actin, due to the high density of astrocytes and their actin-based stellate morphology^25,26,57^, and using an actin inhibitor allowed for direct assessment of the effect of small molecule delivery on the ONH.

## Results

### Local cytochalasin D delivery to the optic nerve reduces optic nerve head filamentous actin content

**Figure 1:**
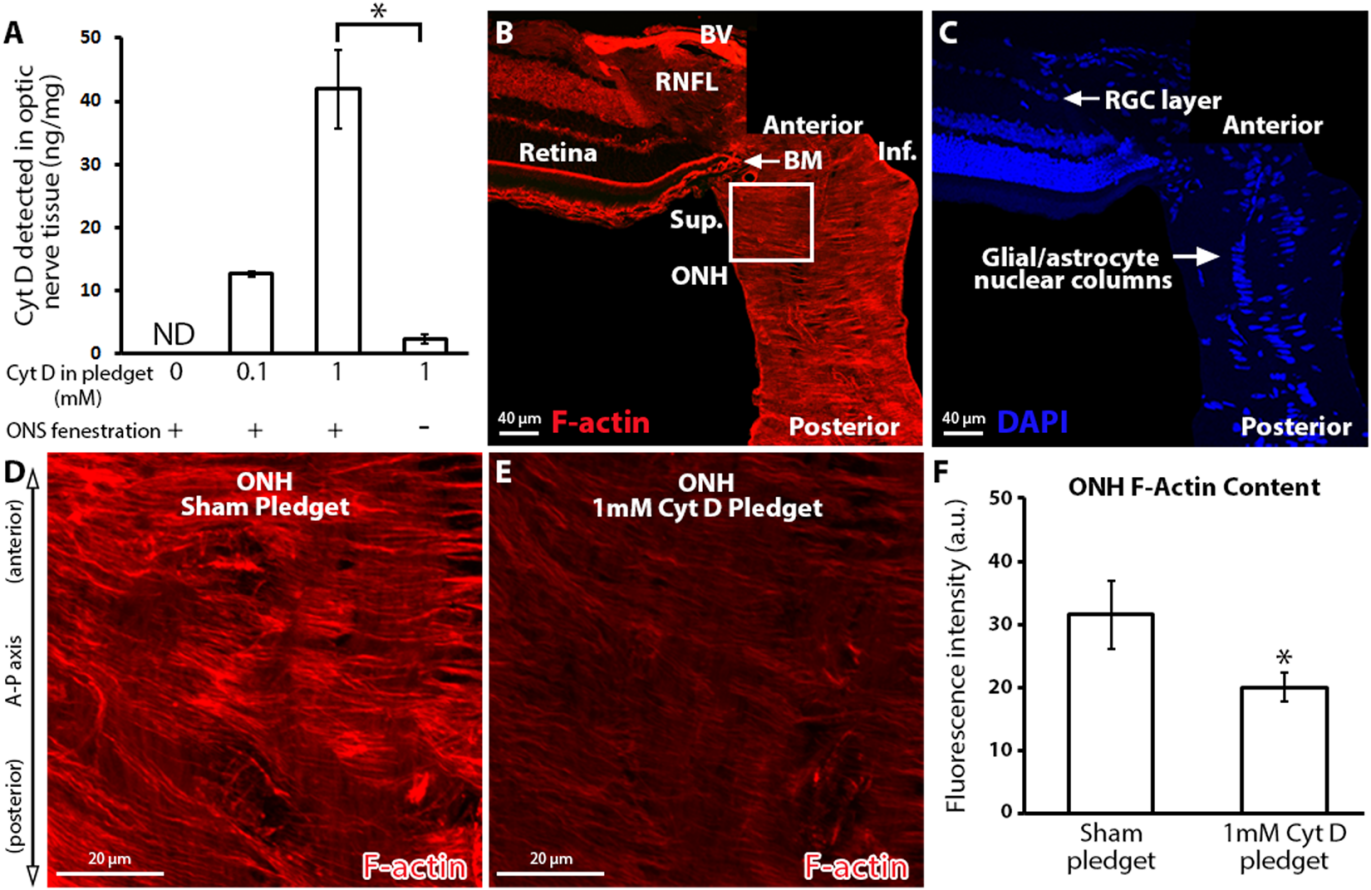
The effect of local cytochalasin D delivery on optic nerve head filamentous actin content. (**A**) Total Cyt D (ng) detected in the anterior 2 mm of optic nerve after surgical delivery, normalized to the total protein (mg) content of the anterior 2 mm optic nerve per animal. Note the significant reduction in Cyt D delivery to the optic nerve in the absence of optic nerve sheath fenestration (n = 2, 2, 3, 3 from left to right). (**B**,**C**) Montage of normal anterior optic nerve and superior retina, labeled with fluorescent-labeled phalloidin (filamentous actin maker) and DAPI (nuclear maker). The superior ONH is identified by the white box. (**D**,**E**) ONH after sham (pledget soaked in vehicle only) delivery and 1 mM Cyt D delivery to the junction of the superior optic nerve and globe. (**F**) ONH F-actin content assessed by fluorescence intensity measurement of fluorescent-labeled phalloidin after sham (n = 4) and 1 mM Cyt D delivery (n = 6). *p < 0.05. Error bars indicate standard error of the mean. A–P = anterior-posterior; a.u. = arbitrary units; BM = Bruch’s membrane; BV = blood vessel; Cyt D = cytochalasin D; F-actin = filamentous actin; Inf. = inferior; ND = none detected; ONH = optic nerve head; ONS = optic nerve sheath; RGC = retinal ganglion cell *layer*; RNFL = retinal nerve fiber layer; Sup. = superior.

We first determined the amount of Cyt D that was delivered to the anterior optic nerve using our surgical technique. The amount of Cyt D detected by LC/MS (ng) was normalized to the total anterior optic nerve tissue protein content analyzed (mg), and was found to depend on the concentration of Cyt D in the pledget that was surgically delivered (Fig. 1A; 12.6 ± 0.3 and 41.9 ± 6.2 ng/mg of Cyt D detected by LC/MS after 0.1 and 1 mM Cyt D in the pledget, respectively). Of note, it was determined that optic nerve sheath fenestration was a critical step in enhancing Cyt D delivery to the anterior optic nerve (Fig. 1A; 41.9 ± 6.2 versus 2.3 ± 0.7 ng/mg of Cyt D detected with and without optic nerve sheath fenestration, respectively; p < 0.05). We attempted to determine the distribution of Cyt D within the optic nerve tissue by using a fluorescent-tagged Cyt D (Cyt D Everfluor) in our surgical approach. However, despite optic nerve fenestration, fluorescent-tagged Cyt D was unable to penetrate the optic nerve tissue and remained at the interface of the optic nerve sheath and optic nerve by microscopic evaluation (Supplemental Fig. 1).

We next determined the local effect of Cyt D on ONH filamentous actin. The ONH is a highly actin-rich and cellular structure (Fig. 1B,C), namely due to the high density and morphology of local astrocytes^25,26^. High magnification of the ONH revealed dense, highly ordered, and actin-rich astrocyte processes after sham pledget delivery (Fig. 1D), consistent with previously reported architecture of the actin-based astrocytes within the ONH^25,26^. After Cyt D delivery, however, ONH filamentous actin labeling was significantly reduced relative to sham delivery (20.0 ± 2.3 versus 31.5 ± 5.4 a.u., respectively, p < 0.05), consistent with reduced total filamentous actin bundle content (Fig. 1D–F).

### Delivery of cytochalasin D to the optic nerve does not affect optic nerve head and retinal ganglion cell *layer* nuclear counts

**Figure 2:**
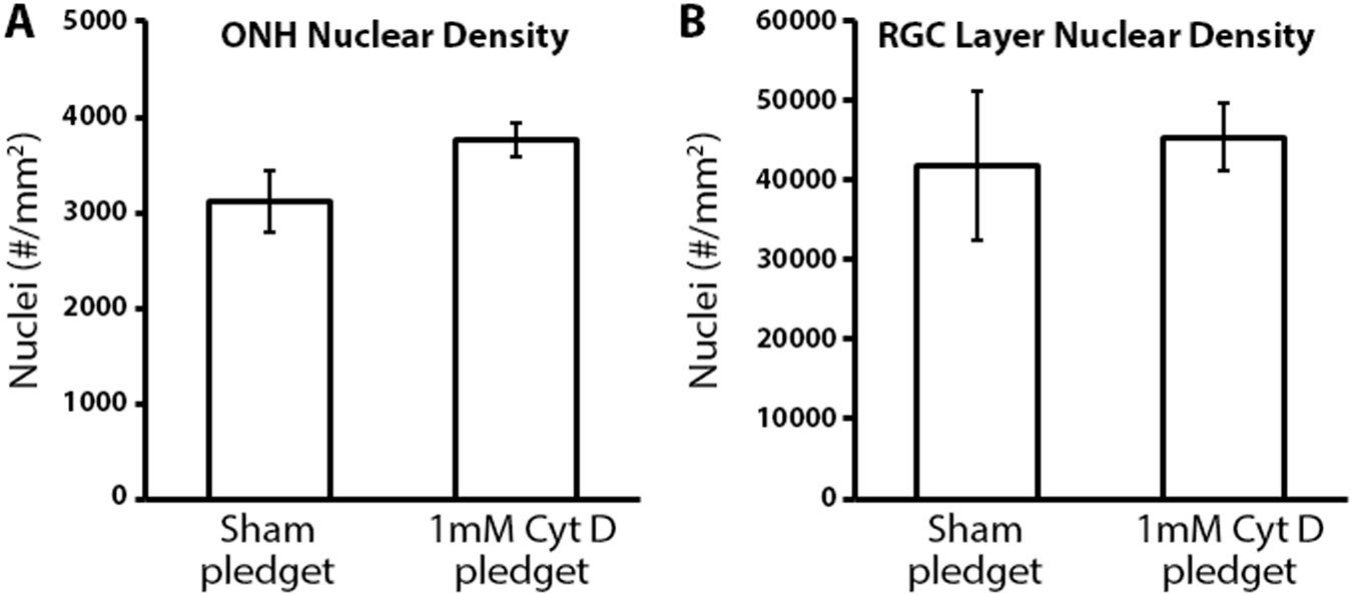
The effect of cytochalasin D delivery on optic nerve head and retinal ganglion cell layer nuclear counts. (**A**) Nuclear density within the anterior optic nerve head (ONH; 5 µm sections; 0–100 µm posterior to Bruch’s membrane) after sham (n = 4) and cytochalasin D (Cyt D; n = 7) delivery to the optic nerve (no statistically significant difference was noted between groups). (**B**) Nuclear density within the retinal ganglion cell (RGC) *layer* of the superior peripapillary retina (5 µm sections; 0–250 µm from the superior optic nerve head) after sham (n = 4) and Cyt D (n = 6) delivery to the optic nerve (no statistically significant difference was noted between groups). Error bars indicate standard error of the mean.

We asked if the observed reduction in filamentous actin within the ONH after cytochalasin D delivery was in fact due to filamentous actin reduction or due to loss of cells (e.g. acute cellular toxicity due to Cyt D). The vast majority of nuclei within the ONH are astrocytic in origin^58^, and arranged in glial columns within the ONH (Fig. 1C). We assessed ONH nuclear density after sham and Cyt D delivery as a marker for ONH astrocyte density. Nuclear densities within the ONH after sham and Cyt D delivery to the optic nerve were found to be similar (3,125 ± 324 versus 3,671 ± 164 nuclei/mm^2^, respectively) with no statistically significant difference between groups (Fig. 2A).

Given the high density of retinal ganglion cell (RGC) axons within the ONH^26^, as well as the close proximity of the superior peripapillary retina to the site of Cyt D delivery (Fig. 1B,C), we asked if Cyt D delivery to the optic nerve led to RGC *layer* cell loss within the retina. Retinal ganglion cell bodies make up at least 50% of the total nuclei within the RGC *layer* of the rat retina^59^, and any adverse global effects of Cyt D on RGCs is likely to cause significant RGC *layer* nuclear loss. Nuclear densities (as assessed by DAPI labeling) of the superior peripapillary RGC *layer* after sham and Cyt D delivery were found to be similar (41,800 ± 9,354 versus 45,333 ± 4,221 nuclei/mm^2^, respectively) with no statistically significant difference between groups (Fig. 2B).

### Cytochalasin D delivery to the optic nerve does not affect optic nerve head axonal microtubule levels

**Figure 3:**
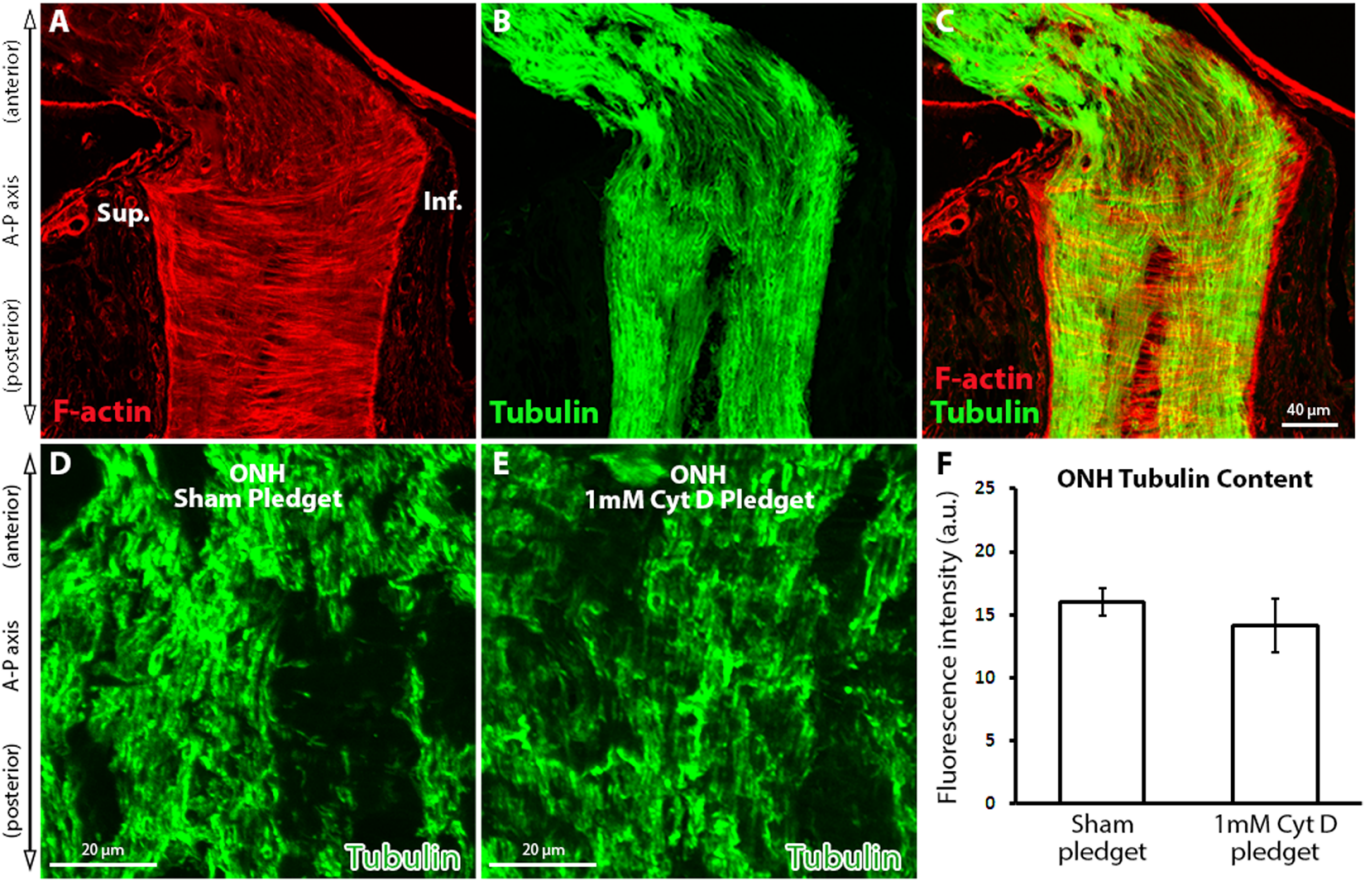
The effect of cytochalasin D delivery on optic nerve head axonal microtubule levels. (**A**–**C**) Normal optic nerve head (ONH) labeled with fluorescent-tagged phalloidin (F-actin maker) and axon-specific anti-βIII tubulin antibodies. (**D**,**E**) ONH tubulin levels after sham (pledget soaked in vehicle only) delivery and 1 mM Cyt D delivery to the junction of the superior optic nerve and globe. (**F**) ONH tubulin content assessed by fluorescence intensity measurement of anti-βIII tubulin antibody labeling after sham (n = 4) and 1 mM Cyt D delivery (n = 6) to the optic nerve (no statistically significant difference was noted between groups). Error bars indicate standard error of the mean. a.u. = arbitrary units; Cyt D = cytochalasin D; F-actin = filamentous actin; Inf. = inferior; ONH = optic nerve head; Sup. = superior.

Axons within the ONH are rich in microtubule filaments, which traverse the nerve perpendicular to the actin filament bundle orientation (Fig. 3A–C). As Cyt D is a highly specific inhibitor of actin assembly, and not microtubule assembly^60^, we asked if Cyt D delivery to the optic nerve had any effects on the microtubule-based axonal cytoskeleton. Axon-specific microtubule labeling within the ONH (using anti-βIII tubulin antibodies) was robust in sham and Cyt D delivery groups (Fig. 3D,E). No statistically significant difference in tubulin label intensity was noted between sham and Cyt D delivery groups (Fig. 3F; 16.0 ± 1.1 versus 14.2 ± 2.1 a.u., respectively).

### Surgical technique for small molecule delivery to the optic nerve causes minimal observable axon injury

We assessed whether the surgical technique and pledget placement alone without small molecular delivery (i.e. sham pledget placement only) resulted in any observable optic nerve axon injury. Ten days post sham pledget placement, no statistically significant optic nerve axon injury was observed relative to contralateral, non-surgical optic nerves using a grading scale of 1–5^61,62^ (mean injury grade 1.55 ± 0.27 [n = 10] versus 1.01 ± 0.01 [n = 10], respectively). However, more variability in axon degeneration (signified by the relatively larger standard error of the mean) and a trend toward higher axon injury was noted in the pledget only group relative to non-surgical optic nerves.

## Discussion

The exact mechanisms of glaucomatous axon injury within the optic nerve remain elusive, and the mainstay of treating glaucomatous eyes (namely IOP reduction^1,63^) does not yet include targeted modulation of cellular function within the retina, ONH, or optic nerve. Furthermore, testing of potential axon- and glia-protective small molecules that act at the level of the ONH and optic nerve to preserve and/or restore axons has been limited by the lack of a delivery method in *in vivo* experimental models of IOP elevation and glaucoma^29^. To address this, we developed a technique for small molecule delivery to the optic nerve in a rodent model. We used our novel surgical approach to deliver a small molecule actin inhibitor (Cyt D) to the optic nerve. Next, we developed an assay for small molecule detection and quantification of Cyt D in optic nerve tissue matrix using LC/MS. Lastly, in proof-of-principle experiments, we demonstrated the reduction in total filamentous actin labeling within the ONH after Cyt D delivery *in vivo*.

This new surgical technique for small molecule delivery to the optic nerve *in vivo* has several highlights. The technique specifically visualizes the site of small molecule delivery (namely the superior junction of the globe and optic nerve), with the addition of creating an optic nerve sheath fenestration, which enhances small molecule delivery to the optic nerve tissue (Fig. 1A). Wang *et al*. reported a similar surgical technique of optic nerve exposure in rats (although without optic nerve sheath fenestration) for the delivery of the small peptide endothelin-1^54^. While delivery of endothelin-1 to the optic nerve tissue was not quantitatively and directly confirmed by Wang *et al*., the effect of endothelin-1 on axonal transport in the optic nerve was observed^54^. Future studies using our technique may confirm that the additional step of optic nerve sheath fenestration would also enhance delivery of small peptides to the optic nerve. Lingor *et al*. have shown direct effects of small interfering RNA on retinal expression of target protein after small interfering RNA delivery to the optic nerve stump post axotomy^55^. This further supports the notion that disruption of the optic nerve sheath is likely important in enhancing small molecule delivery to the optic nerve tissue. Next, our surgical technique is complemented by a newly described method for optic nerve tissue homogenization and analytical detection of small molecule delivery to the optic nerve tissue using LC/MS. Furthermore, the technique allows for local delivery of small molecules for cellular modulation and/or pharmacologic testing, which may otherwise have negative effects if delivered through a systemic, intrathecal, or intravitreal route. Lastly, the surgical approach may also be used to deliver small molecules to the posterior ocular sclera, or the retina (if coupled with scleral fenestration). Such an approach may be useful to research taking place currently to examine the contribution of scleral mechanics and modulation in glaucoma models^64–67^.

While Cyt D is a highly specific actin filament depolymerizer^56^, it is not cell specific. Thus, all cells within the optic nerve are exposed to Cyt D after local delivery through our technique. The majority of actin within the ONH is astrocyte derived^25^, but we could not prevent exposure of other ONH cells and tissue (namely axons and blood vessels) to Cyt D. Thus, while we selected Cyt D for our proof-of-principal small molecule delivery experiments given the highly actin-rich ONH environment, we would not promote Cyt D delivery to the ONH as a therapeutic or even investigational tool in glaucoma models, per se. However, our surgical technique may be used to deliver cell-specific inhibitors, including astrocyte-specific modulators, which are preferentially taken up by glial cells and not by axons^68^.

The surgical technique described here does have some limitations. First, while drug delivery is relatively local to the site of pledget placement (compared to systemic, intrathecal, or intravitreal delivery), the small molecule presumably does gain access to the entirety of the retrobulbar and intraconal space within the orbit. Second, the surgical technique does require surgical experience and knowledge of the ocular and orbital anatomy in a rat model. Third, while no statistically significant difference was noted in observable axon injury after pledget placement only (i.e. sham surgery) relative to non-surgical fellow eyes, the surgical technique (or the pledget itself) does seem to cause a trend toward axon injury in our study. In addition, the standard error of the mean is larger for the surgical group relative to non-surgical fellow eyes, indicating that there is some technique variability from animal to animal, which may cause some axon injury in a variable fashion. Additional experiments with this technique, as well as future modifications designed to further reduce inadvertent nerve trauma, are likely to reduce axon injury further.

While we’ve quantitatively demonstrated delivery of Cyt D to the optic nerve tissue using LC/MS, it’s unclear how uniformly the Cyt D is distributing within the ONH and optic nerve tissue. In related experiments, we attempted to deliver Cyt D modified with a fluorescent tag (Cyt D Everfluor) to visualize the distribution of Cyt D within the optic nerve tissue after delivery (Supplemental Fig. 1). However, due to changes in the molecular weight of Cyt D after the addition of the fluorescent tag (Cyt D MW = 507.62, relative to Cyt D Everfluor MW = 887.81), as well as the presumed changes in solubility and permeability properties after modification with a fluorescent molecule, the fluorescent-tagged Cyt D was unable to penetrate the optic nerve tissue, and remained at the interface of the optic nerve sheath and optic nerve tissue (Supplementary Fig. S1). Lastly, the inclusion of an optic nerve sheath fenestration, while facilitating the delivery of small molecules to the optic nerve tissue, may result in lowering intracranial cerebral spinal fluid pressure (ICP), at least locally at the site of the ONH. Lower ICP has been associated with glaucomatous progression^69,70^, and may also contribute to the slight trend toward more axonal injury in the pledget only sham group relative to fellow eyes in our study. Additional studies of this technique, in combination with IOP elevation to induce glaucomatous damage^37^ may shed further light on this issue.

## Materials and Methods

All animals were treated in accordance with the Association for Research in Vision and Ophthalmology statement for the use of animals in ophthalmic and vision research^71^ and all experimental methods were approved by the Oregon Health & Science University Institutional Animal Care and Use Committee. All data generated or analyzed during this study are included in this published article (and its Supplementary Information files).

### Animals and surgical technique for cytochalasin D delivery to the ONH

**Figure 4:**
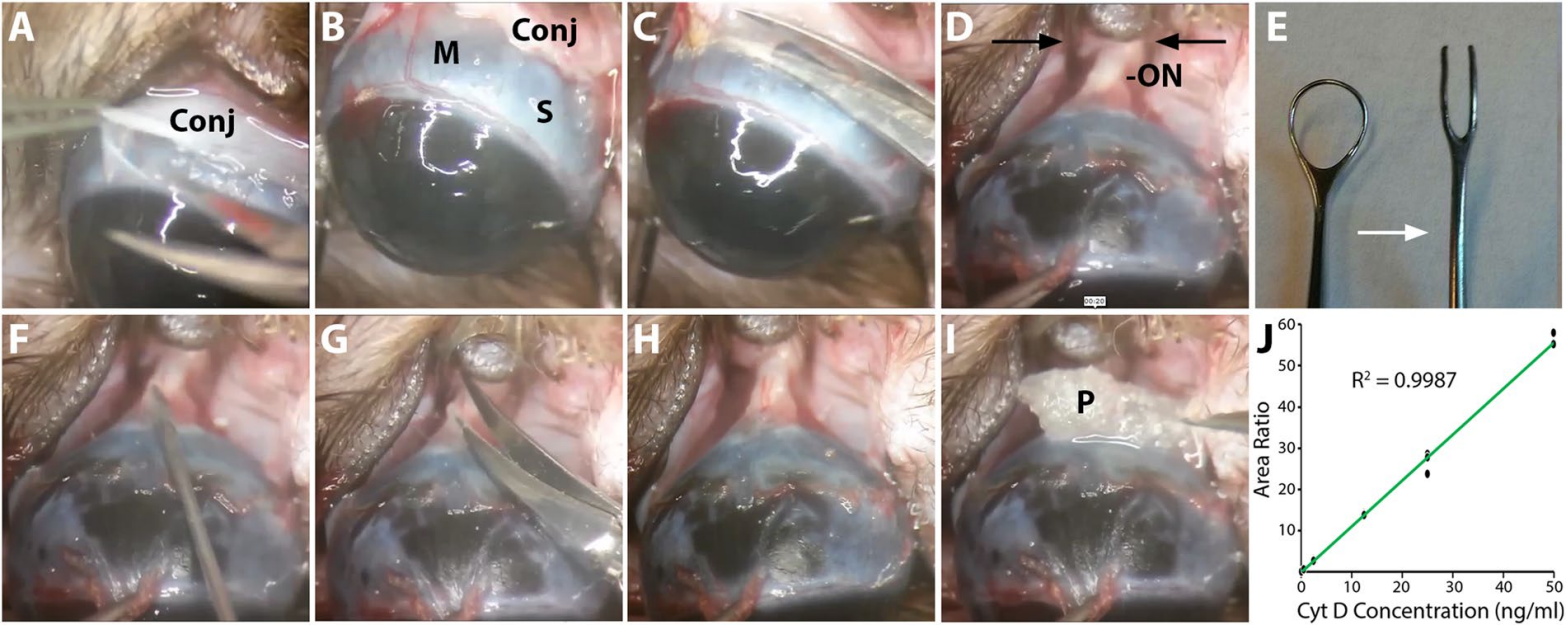
Surgical delivery and analytical detection of cytochalasin D in optic nerve tissue in rats. Superior conjunctival dissection (**A**) allowed for visualization of the superior rectus muscle insertion (**B**) which was cauterized and resected (**C**). The globe was infra-ducted and orbital contents were held posteriorly using a two-pronged instrument (black arrows) to reveal the optic nerve (D). The two-prong instrument was modified from a Lewis Lens Loop by cutting the distal portion of the Lens Loop, straightening the resulting prongs, and smoothing the sharp edges with 800 grit silicon carbide abrasive paper (E; white arrow indicates transition from pre- and post-modification). An MVR blade was used to pierce the optic nerve sheath (**F**), followed by optic nerve sheath fenestration with blunt-tipped Vannas scissors (**G**). The optic nerve was visualized (**H**) and a pledget soaked in vehicle or cytochalasin D (Cyt D) was placed at the junction of the optic nerve and globe (**I**). Standard curve for Cyt D detection in optic nerve tissue matrix using liquid chromatograph/mass spectrometry; triplicates of Cyt D at 0, 0.05, 0.5, 2.5, 12.5, 25, and 50 ng/ml are shown (**J**). Conj = conjunctiva; M = muscle (superior rectus); ON = optic nerve; P = pledget; S = sclera.

Thirty-one 8–9 month-old Brown Norway rats (350–400 g) underwent systemic anesthesia with intraperitoneal injection of ketamine (37.5 mg/kg; JHP Pharmaceuticals, Rochester, MI, USA), xylazine (7.5 mg/kg; RXV, Greeley, CO, USA), and acepromazine maleate (1.5 mg/kg; VET ONE, Boise, ID, USA). Topical 0.5% proparacaine hydrochloride ophthalmic solution (Akorn, Lake Forest, Illinois, USA) was applied to the ocular and conjunctival surface unilaterally. During anesthesia, animals remained on a temperature-controlled blanket at 37 °C to maintain body temperature. Unless otherwise noted, all surgical instruments were purchased from Storz Ophthalmic Instruments (St. Louis, MO, USA). The unilateral surgical technique described below is summarized in a surgical video (Supplementary Video S2). The lateral canthus was clamped with a Mosquito clamp, followed by a lateral canthotomy with Wescott scissors to improve access to the globe. The eye was infero-ducted by placing an eyelid speculum inferiorly. A peritomy and conjunctival dissection was performed at the superior six clock hours of the limbus (Fig. 4A). After careful dissection of the subconjunctival adhesions, the conjunctiva was retracted by an assistant to expose the superior rectus muscle insertion near the limbus (Fig. 4B). To gain access to the intraconal retrobulbar space, the superior rectus muscle was cauterized with low-temp cautery (Bovie Medical, Clearwater, FL, USA) and incised with Vannas scissors along the site of cautery to avoid excess bleeding (Fig. 4C). Care was taken to avoid injury to the superotemporal and superonasal vortex veins exiting the sclera at the mid-equator of the globe. The eye was then further infero-ducted by grasping the superior rectus muscle insertion using 0.12 toothed forceps (Fig. 4D). The junction of the superior optic nerve and the globe was visualized using a two-pronged instrument modified from a Lewis Lens Loop (Storz, E648; Fig. 4D,E), with care to avoid any trauma or excess stretch to the optic nerve. Next, an MVR blade was used to carefully puncture the optic nerve sheath at the superior junction of the optic nerve and the globe (Fig. 4F), with care to minimize injury to the underlying neural optic nerve tissue. This was followed by optic nerve sheath fenestration of the superior optic nerve sheath, using a second pair of Vannas scissors with blunted tips to avoid damage to the underlying optic nerve tissue (Fig. 4G). After adequate optic nerve sheath fenestration was confirmed with visual inspection (Fig. 4H), a 1.5 mm core of Surgifoam pledget (a biocompatible and biodegradable gelatin-based foam; Johnson & Johnson Wound Management, Neenah, WI, USA) soaked either in vehicle (10% dimethyl sulfoxide[DMSO] in phosphate-buffered saline [PBS]) or 0.1–1 mM Cyt D (Sigma-Aldrich, Saint Louis, MO, USA) in 10% DMSO/PBS was placed at the superior junction of the optic nerve and globe (Fig. 4I). The orbital contents and conjunctiva were carefully replaced in their original position, and the conjunctiva was re-approximated at the limbus using 2 interrupted 10-0 Nylon sutures (Medtronic, Minneapolis, MN, USA). The periocular surface was cleaned and erythromycin ointment (Perrigo, Minneapolis, MN, USA) was applied to the ocular surface. Animals received post-surgical subcutaneous injections of 0.05–1.0 mg/kg buprenorphine (Buprenex, Reckitt Benckiser Pharmaceuticals, Richmond, VA, USA) for analgesia and were allowed to awaken from anesthesia on a warm platform. The surgery for each animal from conjunctival peritomy to conjunctival closure lasted approximately 10–15 minutes. To visually inspect the delivery of Cyt D to the optic nerve tissue, 3 additional animals underwent the above surgical technique in which Cyt D was substituted with fluorescent-tagged Cyt D (Cyt D Everfluor BODIPY TMR conjugate, Setareh Biotech, Eugene, OR, USA). One of the animals above received direct injection of Cyt D Everfluor solution into the optic nerve as a positive control for Cyt D Everfluor visualization in prepared tissue.

### Analytical quantification of cytochalasin D delivery to the optic nerve

Animals (n = 10) were anesthetized and sacrificed 4 hours post-surgery for analytical quantification of Cyt D delivery to the optic nerve. Fresh optic nerve tissue (2 mm of total nerve tissue measured from the juncture with the sclera) was collected on ice, rinsed with PBS to wash off any Cyt D on the surface of the tissue, frozen on dry ice, and stored at −80 °C until ready for the following steps. Optic nerve tissue was suspended in 110 µl of PBS and homogenized using a compact lab homogenizer (Hielsher UP50H, Teltow, Germany). Ten µl were taken from each homogenized sample for total protein content analysis, while the remaining 100 µl of sample was stored at −80 °C until ready for processing for Cyt D quantification by the Bioanalytical Shared Resource/Pharmacokinetics Core (Oregon Health & Science University, Portland, Oregon, USA). For total protein content analysis, the 10 µL aliquot of optic nerve homogenate was centrifuged at 13,000 rpm at 4 °C for 10 minutes; 2 µL of the supernatant was used to quantify total protein content using the Nanodrop 2000 spectrophotometer (ThermoFisher scientific, Waltham, MA, USA).

Cyt D and Cyt B (Sigma-Aldrich; Cyt B was used as an internal standard in LC/MS analysis) were analyzed adopting the methods described by Prasain *et al*.^72^ and Evidente *et al*.^73^. Optic nerve homogenate was thawed at room temperature, transferred to 13 × 100 screw cap glass tubes and diluted for ease of handling with 0.9 ml of PBS. The diluted homogenate was spiked with 0.5 ng of the internal standard (Cyt B in DMSO). The samples were then extracted twice with 2 ml of ethyl acetate. The combined organic phases were dried under vacuum for ~50 min at 40 °C using a Speedvac concentrator. Dried extracts were then dissolved in 50 µl of ethanol with 10% DMF and 10 mM ammonium acetate, sonicated for 1 minute and then placed into sample vials. Sample vials were placed into the autosampler maintained at 35 °C and pre-heated for 15 minutes prior to sample injection. Calibrators were prepared by spiking 0, 0.05, 0.5, 2.5, 12.5, 25, and 50 ng/ml of Cyt D in sample equivalents of PBS and extracted at the same time as the samples (Fig. 4J). Two quality control samples of homogenate at 0.05 ng/ml and 50 ng/ml were prepared and extracted along with samples. The samples were analyzed by LC/MS, with a 10 µl injection volume.

Cyt D and Cyt B were analyzed using an Applied Biosystems QTRAP 4000 instrument (Foster City, CA, USA) with electrospray ionization in positive mode. The mass spectrometer was interfaced to a Shimadzu (Columbia, MD, USA) SIL-20AC XR auto-sampler followed by 2 LC-20AD XR LC pumps. The instrument was operated with the following settings: source voltage 4500 kV, GS1 50, GS2 50, CUR 10, TEM 650, and CAD MEDIUM. The scheduled multiple reaction monitoring (MRM) transitions monitored are listed in Table 1. Compounds were infused individually and instrument parameters optimized for each MRM transition. A gradient mobile phase was delivered at a flow rate of 0.2 ml/min, and consisted of two solvents A: 0.05% acetic acid in water and B: 0.05% acetic acid in acetonitrile. Separation was achieved using a Gemini-NX 100 × 2 mm, 3 µ column (Phenomenex, Torrance, CA, USA) at 40 °C and the autosampler was kept at 35 °C which was critical for preventing the precipitation of Cyt B internal standard. The gradient elution was as follows: initial %B of 10%, held for 1 min; increase to 100% B at 10 min; 100% B was held for 2 min; decrease to 10% B over 0.1 min; hold at 10% B for 3.2 minutes to re-equilibrate. Data were acquired using Analyst 1.5.1 software and analyzed using Multiquant 2.1.1.

**Table 1:** Scheduled MRM transitions monitored for mass spectrometry quantification of cytochalasin D.

Compound	Retention Time (min)	Q1 Mass	Q3 Mass	DP (volts)	EP (volts)	CE (volts)	CXP (volts)
**Cyt D**	**8.3**	**530**	**470**	**145**	**10**	**41**	**6**
Cyt D	8.3	530	91	145	10	127	4
**Cyt B ISTD**	**8.3**	**502.2**	**328**	**141**	**10**	**39**	**6**
Cyt B ISTD	8.3	502.2	484	141	10	45	10

Cyt B/D = cytochalasin B/D; CE = collision energy; CXP = collision cell exit potential; DP = declustering potential; EP = entrance potential; ISTD = internal standard; LC/MS = liquid chromatography/mass spectrometry; MRM = multiple reaction monitoring; Q = quadrupole; bold = used for quantification.

### Actin labeling and immuno-labeling of optic nerve and retinal tissue

In order to determine the effect of local Cyt D delivery on ONH tissue, animals (n = 11) were anesthetized and sacrificed 24 hours post Cyt D pledget placement by transcardial perfusion fixation with freshly prepared buffered 4% formaldehyde solution. Perfusion-fixed eyes (including the ONH) were cryopreserved in 15% sucrose/PBS, followed by 30% sucrose/PBS, positioned for vertical longitudinal sectioning in 1:1 solution of Optimal Cutting Temperature support medium (Sakura Finetek, Torrance, CA, USA) and 30% sucrose/PBS, frozen in liquid isopentane cooled by liquid nitrogen, and cryo-sectioned (5 µm) onto glass slides. The superior and inferior orientation of the ONH was determined based on the anatomic location of the central retinal vein, located just inferior to the ONH^74^. Tissue sections were blocked with 1% bovine serum albumin (BSA) in PBS for 1 hour at room temperature. Actin bundles were labeled with tetramethylrhodamine (TRITC)-labeled phalloidin (Sigma-Aldrich) at a concentration of 0.1 mg/ml in PBS at room temperature for 1 hour^25,75^, followed by three cycles of wash with PBS at room temperature for 5 minutes. Tissue sections were co-labeled with primary antibodies in 1% BSA/PBS at 4 °C overnight, using antibodies against axonal tubulin (Tuj1; mouse monoclonal against βIII tubulin, Covance, Seattle, WA, USA). Sections were washed as above, followed by incubation with secondary Alexa 488-labeled goat anti-mouse monoclonal antibodies (ThermoFisher Scientific) in 1% BSA/PBS at 4 °C for 1 hour at room temperature. After washing as above, cell nuclei were stained with the mounting media (Prolong Gold with DAPI, ThermoFisher Scientific).

### Axonal injury assessment

To determine any potential optic nerve axon injury due to the surgical technique alone, a group of additional rats (n = 10) underwent the above surgical approach unilaterally using pledget soaked in vehicle buffer only. The animals were allowed to recover from surgery and were sacrificed 10 days post pledget placement (equivalent to the time necessary for essentially complete axon degeneration after optic nerve transection^25^). The retrobulbar optic nerves (including the contralateral fellow eyes which did not undergo surgical intervention) were perfusion-fixed with 4% paraformaldehyde, followed by 5% glutaraldehyde, embedded in plastic, transversely sectioned and stained with toluidine blue (Electron Microscopy Sciences, Hatfield, PA, USA), followed by light microscopy grading for morphologic axonal degeneration on a scale of 1 (no axonal injury) to 5 (axonal degeneration involving the entire nerve area), as previously described^61,62^. Sections were masked and injury was graded by 6 observers and the grades were averaged.

### Microscopy and image analysis

Prior to confocal imaging, slides were labeled with a unique 4-digit number. Next, random samples were briefly used to determine the saturation and background intensity levels of the samples under confocal microscopy and camera settings (FV1000 microscope [Olympus, Center Valley, PA, USA] and an UplanFLN 40×/NA1.30 oil objective). Using the Intensity Mode setting of the confocal microscope and camera, we made certain that the intensities being captured were within the linear range of the camera to avoid over and under saturation. These settings were then applied to all samples going forward to maintain consistent imaging. Confocal images of the superior ONH (defined as the superior half of the ONH 0-100 µm posterior to the termination of Bruch’s membrane after longitudinal, vertical sectioning centered on the optic nerve) and the superior retina (defined as 250 µm of the superior peripapillary retina after longitudinal, vertical sectioning centered on the optic nerve) were obtained. Images were captured using FV10-ASW version 4.0 software (Olympus) with laser wavelength settings of 405 nm, 488 nm, and 559 nm at 1 µm/slice. Confocal microscopy image acquisition was performed in the same manner on all slides (including the same laser settings, magnification, exposure time, intensity range, and image capture size). Captured images were imported directly into FIJI image analysis software (http://fiji.sc/Fiji; an open source image processing package based on the National Institute of Health [Bethesda, Maryland, USA] software ImageJ: http://imagej.nih.gov/ij/), and were analyzed as Z-stacks in a semi-automated fashion (including analyzing the entire Z-stack image at once without any user manipulation, and using the same analysis settings for all images). Mean fluorescence pixel intensities were calculated using FIJI software. Upon completion of image acquisition and analysis, the key for identifying which sample/data belonged to which treatment group was then used to group the samples. For RGC *layer* nuclei quantification, nuclear counts were normalized to the same areas of superior peripapillary retina (0.00125 mm^2^), using our combined retina and optic nerve sections, which were vertically-sectioned along the central, longitudinal axis of the optic nerve within each sample (Fig. 1B,C). Statistical analysis was performed by 2-tailed, 2-sample of equal variance t-test and one-way analysis of variance (ANOVA) (GraphPad Prism software, La Jolla, CA, USA).

## Electronic supplementary material

Supplementary Figure S1

Supplementary Video S2
