# Supplementary material for: In Vivo Small Molecule Delivery to the Optic Nerve in a Rodent Model
Source: Sci Rep. 2018 Mar 13;8:4453. doi: 10.1038/s41598-018-22737-4 (PMC5849600; doi:10.1038/s41598-018-22737-4)
Supplement: Supplementary file 1 — Supplementary Figure S1 [file 41598_2018_22737_MOESM1_ESM.pdf]

In Vivo Small Molecule Delivery to the Optic Nerve in a Rodent Model

Shandiz Tehrani*, R. Katherine Delf, William O. Cepurna, Lauren Davis, Elaine C. Johnson, John C. Morrison

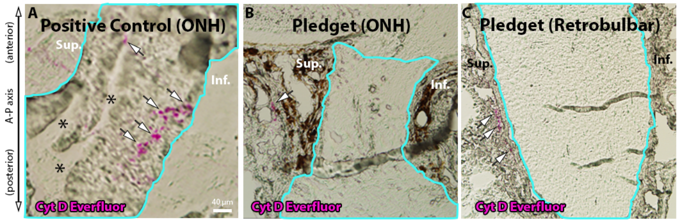
A
Positive Control (ONH)
Sup.
Inf.
(anterior)
A-P axis
(posterior)
Cyt D Everfluor
40 µm
B
Pledget (ONH)
Sup.
Inf.
Cyt D Everfluor
C
Pledget (Retrobulbar)
Sup.
Inf.
Cyt D Everfluor

SUPPLEMENTARY INFORMATION LEGENDS

Supplementary Figure S1. Fluorescent-tagged cytochalasin D does not significantly penetrate the optic nerve tissue after surgical delivery to the optic nerve. (A) Fluorescent-tagged cytochalasin D (Cyt D Everfluor) visualized within the optic nerve head (ONH) tissue after direct injection into the optic nerve as a positive control for Cyt D Everfluor detection (i.e. without pledget use). Note the multiple areas of Cyt D Everfluor within the ONH tissue (arrows). The tract from the 31g needle injection into the optic nerve tissue is denoted by the asterisks (*). (B) No significant Cyt D Everfluor is observed within the ONH tissue after pledget delivery of Cyt D Everfluor. Note the Cyt D Everfluor signal outside of the ONH tissue (arrow). The perineural brown pigment is typical in pigmented Brown Norway rat tissue. (C) No significant Cyt D Everfluor is observed within the retrobulbar optic nerve tissue after pledget delivery of Cyt D Fluor. Note the Cyt D Everfluor signal outside of the ONH tissue (arrow). Cyt D = cytochalasin D; Inf. = inferior; ONH = optic nerve head; Sup. = superior. Optic nerve tissue is outlined in turquoise.
